# Mitochondrial transfer from Adipose stem cells to breast cancer cells drives multi-drug resistance

**DOI:** 10.1186/s13046-024-03087-8

**Published:** 2024-06-14

**Authors:** Vitale Del Vecchio, Ayesha Rehman, Sameer Kumar Panda, Martina Torsiello, Martina Marigliano, Maria Maddalena Nicoletti, Giuseppe Andrea Ferraro, Vincenzo De Falco, Rosamaria Lappano, Eva Lieto, Francesca Pagliuca, Carlo Caputo, Marcella La Noce, Gianpaolo Papaccio, Virginia Tirino, Nirmal Robinson, Vincenzo Desiderio, Federica Papaccio

**Affiliations:** 1 Department of Experimental Medicine, University of Campania “Luigi Vanvitelli”, Via L. Armanni, 5, 80138 Naples, Italy; 2 Center for Cancer Biology, University of South Australia and SA Pathology, Adelaide, Australia; 3 Department of Medicine, Surgery and Dentistry “Scuola Medica Salernitana”, Via Salvador Allende, 43, Baronissi, Sa Italy; 4 Unit of Dermatology, Department of Mental, Physical and Preventive Medicine, University of Campania “Luigi Vanvitelli”, Via L. De Crecchio, 6, 80138 Naples, Italy; 5 Plastic and Reconstructive Surgery Unit, Multidisciplinary Department of Medical, Surgical and Dental Sciences, University of Campania “Luigi Vanvitelli”, Via L. De Crecchio, 6, 80138 Naples, Italy; 6 Department of Pharmacy, Health and Nutritional Sciences, University of Calabria, Via Pietro Bucci, Arcavacata di Rende, 87036 CS Italy; 7 Department of Translational Medicine, University of Campania “Luigi Vanvitelli” Via Leonardo Bianchi, 80131 Naples, Italy; 8 Department of Mental, Physical and Preventive Medicine, University of Campania “Luigi Vanvitelli”, Largo Madonna delle Grazie n. 1, 80138 Naples, Italy; 9 Department of Precision Medicine, University of Campania “Luigi Vanvitelli”, Via De Crecchio, 7 - 80138 Naples, Italy; 10 Unit of Cytometry and Mutational Diagnostics, AOU “Luigi Vanvitelli”, Napoli, Italy

**Keywords:** Mitochondrial transfer, Tunneling nanotubes, Mitoception, Adipose Stem cells, Multi-drug resistance, Breast Cancer

## Abstract

**Background:**

Breast cancer (BC) is a complex disease, showing heterogeneity in the genetic background, molecular subtype, and treatment algorithm. Historically, treatment strategies have been directed towards cancer cells, but these are not the unique components of the tumor bulk, where a key role is played by the tumor microenvironment (TME), whose better understanding could be crucial to obtain better outcomes.

**Methods:**

We evaluated mitochondrial transfer (MT) by co-culturing Adipose stem cells with different Breast cancer cells (BCCs), through MitoTracker assay, Mitoception, confocal and immunofluorescence analyses. MT inhibitors were used to confirm the MT by Tunneling Nano Tubes (TNTs). MT effect on multi-drug resistance (MDR) was assessed using Doxorubicin assay and ABC transporter evaluation. In addition, ATP production was measured by Oxygen Consumption rates (OCR) and Immunoblot analysis.

**Results:**

We found that MT occurs via Tunneling Nano Tubes (TNTs) and can be blocked by actin polymerization inhibitors. Furthermore, in hybrid co-cultures between ASCs and patient-derived organoids we found a massive MT. Breast Cancer cells (BCCs) with ASCs derived mitochondria (ADM) showed a reduced HIF-1α expression in hypoxic conditions, with an increased ATP production driving ABC transporters-mediated multi-drug resistance (MDR), linked to oxidative phosphorylation metabolism rewiring.

**Conclusions:**

We provide a proof-of-concept of the occurrence of Mitochondrial Transfer (MT) from Adipose Stem Cells (ASCs) to BC models. Blocking MT from ASCs to BCCs could be a new effective therapeutic strategy for BC treatment.

**Supplementary Information:**

The online version contains supplementary material available at 10.1186/s13046-024-03087-8.

## Background

Breast cancer (BC) is the second most prevalent cancer in the female population worldwide. The treatment algorithm of both early and advanced BC still includes chemotherapy in many therapeutic settings [1, 2]. However, many patients do not respond due to primary or acquired resistance [3].

BC cells (BCCs) are surrounded by mammary adipose tissue and intermingled with a repertoire of stromal cells such as adipose stem cells (ASCs), mesenchymal stem cells (MSCs), cancer-associated fibroblasts (CAFs) with endothelial and immune cells, constituting BC microenvironment (BCME), deeply influencing disease development, progression and treatment response [4]. Interestingly, the adipose component is altered in BC patients, due to strong immune cells infiltration and chronic inflammatory status [5]. MSCs/ASCs play a dominant role in reshaping BCME, promoting epithelial-to-mesenchymal transition (EMT) and supporting cancer stem cells (CSCs), which are, in turn, associated with multi-drug resistance (MDR) [6]. The role of ASCs in MDR was highlighted in a recent study where breast adipose tissue-derived ASCs showed a higher potential to enrich CSCs in BC, that, in turn, led to drug resistance [7].

Cancer cells and MSCs/ASCs can communicate through several mechanisms, such as tunneling nanotubes (TNTs), cell–cell fusion and extracellular vesicles (EVs) trafficking [8]. These enable cells to exchange various intra-cellular components, including macro-molecules, organelles, vesicles, proteins, calcium ions and others [5, 9].

A recent study showed that intercellular mitochondrial transfer (MT) in adipose tissue represents a mechanism of cellular communication regulating systemic metabolic homeostasis [10]. Also, MT was reported to influence multiple myeloma features in terms of metabolic switch, reactive oxygen species (ROS) homeostasis and drug sensitivity [11].

One of the major mechanisms responsible for MDR involves adenosine-5’-triphosphates (ATP) binding cassette (ABC) transporters, that can efflux several drugs outside the cell, decreasing intracellular drug concentrations and rendering treatment ineffective. The main efflux transporters involved in BC MDR are ABCB1 (also termed P-glycoprotein, P-gp, or MDR1), multidrug resistance protein 1 (MRP1/ABCC1) and ABCG2 (also termed breast cancer resistance protein BCRP or mitoxantrone resistance protein MXR) [12, 13]. As these transporters are energy-consuming pumps, they demand considerable amounts of ATP. Remarkably, respiration-deficient BCCs can uptake healthy mitochondria from stromal cells, restoring their functional respiration [14].

Of note, a recent study showed that mitochondrial ATP is the main source of energy for drug efflux in chemo-resistant cancer cells [15]. Interestingly, treatment-resistant CSCs are known to be more dependent on oxidative phosphorylation (OXPHOS) and hence have more functional mitochondria and ATP content than their differentiated progeny [16].

These evidences demonstrate a fundamental role of metabolic rewiring in BC, strongly influenced by tumor microenvironment, where stromal cells regulate the consumption and secretion of metabolites, thus modulating the intrinsic metabolic profile of cancer cells [17].

Here, employing cell lines and patient-derived cultures from BC patients, we showed that ASCs transfer their mitochondria to BC cells via MT. Furthermore, we characterized the functional effect of MT, showing that it induces metabolic rewiring and increased ATP production that fuels ABC transporters efflux activity, thereby driving MDR. As a proof-of-concept, we showed that blocking MT restores drug sensitivity, highlighting it as a new potential target for drug development in BC.

## Materials and methods

### Cells and cell cultures

MCF-7, MDA-MB.231 and hASC52telo hTERT cells were purchased from ATCC. The naturally immortalized BC cell line BCAHC-1 was donated by the Pharmacology Department of the University of Calabria [18]. MCF-7 and MDA-MB.231 cells were cultured in DMEM with glutamine, penicillin, streptomycin, and fetal bovine serum (FBS). BCAHC-1 cells were cultured in DMEM/F‐12 with FBS. hASC hTERT were cultured in Mesenchymal Stem Cell Basal Medium for Adipose-derived MSCs (ATCC) with Mesenchymal Stem Cell Growth Kit for Adipose-derived MSCs (ATCC, Manassas, Virginia). For hypoxia condition, where expected, we treated the cells with Cobalt Chloride (CoCl_2_) 100uM (Merck, Milan Italy). All cell lines were kept at 37 °C in a humidified atmosphere with 5% CO2 under mycoplasma-free conditions (checked every three months).

### Adipose stem cells isolation

pdASCs were isolated from subcutaneous adipose tissue from abdomen or breast of consenting healthy female patients (mean age = 37 ± 2.5 years). Tissue was digested with collagenase type I/dispase and cells were phenotypically validated according to our previous article [19]. The cells were incubated at 37 °C under 5% CO_2_ and the medium changed twice a week.

## Generation of patient-derived organoids

The BCC-66 patient-derived organoids (PDOs) were generated from a consenting BC patient following a previously published protocol [20]. Fresh tissue sample, collected in November 2023, underwent enzymatic digestion with Liberase TL and Y-27632. The obtained cells were embedded in basement membrane matrix (BME) and supplemented with complete medium (Supplementary Table 1).

### Next generation sequencing (NGS)

Total DNA was extracted with PureLink® Genomic DNA Mini Kit (BCC-66) and with QIAamp® DNA FFPE Tissue kit (BT-66, from unstained formalin-fixed paraffin-embedded tissue sections). Libraries were prepared with AmpliSeq Cancer Hotspot Panel v2 (Supplementary Table 2), 2X Oncomine™ Focus DNA Assay, Chef-Ready (Supplementary Table 3) and AmpliSeq Custom Gastric Panel by Thermo Fisher (Supplementary Table 4). The NGS was performed with Ion GeneStudio S5 Plus (Thermo, Waltham, USA) and the performance and results statistically evaluated with Torrent Suite Software v.5.18.1 and Integrative Genomics Viewer (IGV v 2.2, Broad Institute). The significant SNVs were analyzed for previously reported hotspot mutations (COSMIC database).

### Mitochondrial transfer co-culture models

CellTracker™ Blue Dye (Thermo, Waltham, USA) MCF-7, MDA-MB.231 and BCAHC-1 cells were co-cultured with MitoTracker™ Green/Red (Thermo, Waltham, USA) pdASCs/hASCs hTERT cells at 2:1 ratio, with or without 0.4 μm pores cell culture inserts for 24 h. For MT inhibition, co-cultures were treated with Antimycin A (100 nM), Cytochalasin B (Cyt-B 2,5 μM), CCCP (5 μM) or MdiVi-1 (10 μM) (all from Merck, Milan, Italy) before fluorescent microscopy and flow cytometry (FACS) analysis.

To generate a 2D/3D hybrid co-culture model, PDOs were trypsinized and plated without BME. After 48 h, having reached a similar volume they were pre-labelled with CellTracker™ Blue Dye and plated on the top of MitoTracker™ Green/Red ASCs, at the same conditions described before.

### MitoCeption (MCP)

For mitochondria isolation, pdASCs/hASCs hTERT were incubated with mitochondria extraction buffer (220 mM Mannitol, 70 mM Sucrose, 10 mM HEPES/KOH pH-7.4, and 1 mM EDTA), with 1 mM phenyl-methyl sulphonyl fluoride (PMSF), 1X HaltT^M^ Protease Inhibitor Cocktail and 0.2% BSA (all from Merck, Milan, Italy) [21]. MCP quality was evaluated with 100 nM MitoTracker™ Green/Red and quantified with Bradford assay. We typically yielded 100 μg/10^6^ ASCs mitochondria. Mitochondria were added on top of MCF-7 and MDA-MB.231 cells (10 μg/ml) [21].

### Fluorescence and confocal microscopy

For MT qualitative evaluation, BCCs (MCF-7, MDA-MB.231 and BCAHC-1) and pdASCs were plated on slide coverslips. After co-culture, they were fixed with 4% paraformaldehyde and stained with Phalloidin-FITC (Thermo Scientific, Rockford, USA), beta actin monoclonal antibody and beta-tubulin polyclonal antibody (Elabscience, Houston, USA). Samples were incubated for 2 h with Chk pAB to Rb IgG FITC (Abcam). Images were captured with EVOS M5000 Cell Imaging System (Thermo Scientific, Rockford, USA).

MT evaluation in the 2D/3D co-culture was performed via confocal microscopy. BCC-66 were plated on slide coverslips and co-cultured as previously described. After incubation, samples were fixed and stained with Phalloidin-FITC. Images were captured on a Carl Zeiss LSM 700 Confocal Laser Scanning Microscope (Zeiss LSM 700, Oberkochen, Germany) through a 63x/1.4 PlanApo immersion objective and analysed for the Z-stach orthogonal view reconstruction with ImageJ software.

For MCP qualitative evaluation, CellTracker™ Blue Dye MCF-7 and MDA-MB.231 were plated on slide coverslips, subjected to MCP, and imaged via confocal microscopy, followed by co-localization statistical analysis.

For ABC transporter evaluation, cells were treated with Doxorubicin (DOX) (Merck, Milan Italy) (1 µM for MDA-MB.231; 2 µM for MCF-7) for 6 h at 37 °C. P-Glycoprotein, ABCG2, and ABCC1 Polyclonal Antibody (all from Elabscience, Houston, USA) were added after blocking and incubated overnight at 4 °C. Cells were washed and incubated with Chk pAB to Rb IgG FITC (Abcam) and stained with Hoechst 33,342, Trihydrochloride, Trihydrate. Samples were imaged on EVOS M5000 Cell Imaging System. All the microscopy assays have been performed three times in triplicate.

### Doxorubicin assay

MCF-7 and MDA-MB.231 sensitivity to DOX was evaluated with the DOX assay^13^, measuring cell MFI (PE fluorochrome) which is directly correlated with DOX cell retention. Cells were previously subjected to MCP and then treated with DOX (1 µM for MDA- MB.231; 2 µM for MCF-7), Verapamil (VER) (5 µM), D-Glucose (D-Glu) (25 mM), and Rotenone (50 µM) (all from Merck, Milan Italy) for 24 h. Samples were imaged at 20X on EVOS or analyzed with FACS CANTO II (BD Biosciences, San Jose, CA). The assay has been performed three times in triplicate.

### Cell viability assay

MCF-7, MDA-MB.231 and BCAHC-1 viability was measured by the colorimetric 3-(4,5-dimethyl-2-thiazolyl)-2,5- diphenyltetrazolium bromide (MTT) assay. Cells were seeded in triplicate in 96-well plates and treated with Cyt-B (0.5-10 µM), Docetaxel (DTX) (50 nM) and Cisplatin (CIS) (10 µM) for 24 h. The cells were then treated with MTT and the absorbance at 540 nm measured using a microplate ELISA reader. The assay has been performed three times.

### Digital holographic microscopy

For kinetic dose–response assay, a Holomonitor M4 microscope (Phase Holographic Imaging AB, Lund, Sweden) was used. Cells were seeded at a density of 10^4^ cells/well and then subjected to MCP before treatment with DOX (1 µM for MDA-MB.231; 2 µM for MCF-7). Cells were continuously monitored for 24 h, in time-lapse mode (every hour), at multiple positions in each well using a high-precision motorized stage. The Holomonitor App suite cell imaging software was used for image analysis, for the evaluation of the cell viability parameters (cell density = #cells/cm^2^; percentage of confluence = %confluence). The results are showed as changes relative to zero time-point, from each area and presented as mean ± SE for all the monitored areas in each well. The assay has been performed three times in triplicate.

### Flow cytometry

pdASCs immunophenotype was periodically characterized with the following antibodies: CD90 (FITC), CD105 (FITC), CD44 (FITC), CD56 (PE Cy7-A), CD29 (PE Cy7-A), CD19 (PECy5.5), HLA-DR (V500), CD324 (PE-A), CD34 (PE-A) and CD45 (APC). Apoptosis was evaluated with BD Pharmingen™ FITC Annexin V Apoptosis Detection Kit (BD, NJ, USA). For ABC transporter expression/activity evaluation, cells were treated for 6 h with DOX (1 µM for MDA-MB.231; 2 µM for MCF-7), then fixed and permeabilized with FIX & PERM Cell Fixation & Cell Permeabilization Kit (BD, NJ, USA). P-Glycoprotein Polyclonal Antibody, ABCG2 and ABCB1 Polyclonal Antibody respectively were added before incubating with secondary antibody Chk pAB to Rb IgG FITC for 1 h at 4 °C, then revealed by FACS with FACS CANTO II. Analysis was performed with FlowJo V10 software. The assay has been performed three times in triplicate.

### Extracellular flux analysis

Oxygen consumption rate (OCR) was analyzed under basal conditions and in response to sequential injections of oligomycin (2 µM), FCCP (2 µM) and rotenone with antimycin A (0.1 µM each) using the Mitochondrial Respiration XF Cell Mito Stress Test. Assays were performed using manufacturer recommended medium (DMEM, 10 mM glucose, 2 mM glutamine, and 1 mM pyruvate, pH 7.4). OCR evaluations were performed with Agilent Seahorse XF Analyzers 24 h after MCP and 2 h after hypoxia. Analysis assay was performed with the Agilent Seahorse Wave Pro software and data analyzed with the Agilent Seahorse Analytics from the Agilent Seahorse XF Analyzer. The assay has been performed three times in triplicate.

### Immunoblot

Proteins extracts were obtained with 2% SDS containing 2 mM PMSF, 10 μg/ml antipain, leupeptin, and trypsin inhibitor, 10 mM sodium fluoride and 1 mM sodium orthovanadate (Merck, Milan Italy) Immunoblotting was performed with HIF1 alpha Polyclonal Antibody (Elabscience, Houston, USA) and Anti-GAPDH (Merck, Milan, Italy). Peroxidase-conjugate anti-rabbit IgG (Santacruz, Dallas, USA) was used for enhanced chemiluminescence (ECL) detection (BioRad, California, USA). The assay has been performed three times.

### Statistical analysis

Cell viability and FACS data were analyzed with Ordinary Two-Way Anova model, adjusted with Bartlett’s test, with Tukey’s multiple comparison test. MCP analysis was performed with Li’s statistical model, based on the evaluation of the intensity correlation quotient (ICQ) [22]. This value was obtained by counting the positive voxels, normalized on the total number of voxels (related to cell confluence). Five view fields were analyzed for each sample. Statistical significance for Holomonitor data was determined by the Ordinary Two-Way Anova model, adjusted with the Sidak multiple comparison test. Unless otherwise specified statistical analysis were performed with GraphPad Prism9. Two-tailed *p* values < 0.05 were considered significant.

## Results

### Patient-derived adipose stem cells transfer their mitochondria to breast *cancer* cells via tunneling nanotubes

**Fig. 1:**
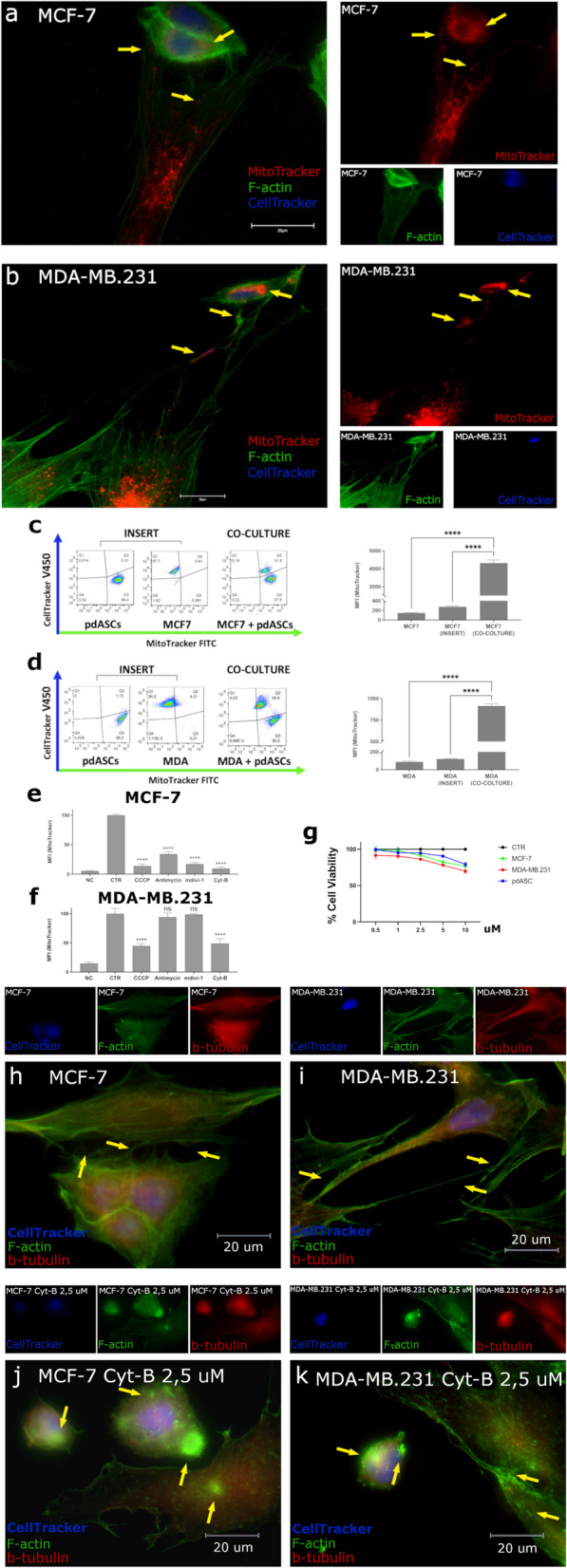
MT occurs between pdASCs and BCCs via TNTs and Actin polymerization inhibition disrupts TNTs inhibiting MT. **a-b** Fluorescence microscopy of pre-labelled MCF7 or MDA-MB.231 (CellTracker-Blue) and pre-stained pdASCs (MitoTracker-Red) with F-Actin (Phalloidin-FITC). The yellow arrows point-out the pdASC mitochondria along the TNTs and into recipient BCCs (100X; Fig. 1b is the merge of two fields of view, required for the capture of the entire TNTs length). **c-d** Flow cytometry analysis of the cell fluorescence, for the quantization of the MT occurring from the pre-stained MitoTracker-FITC pdASCs to the pre-labelled CellTracker-Blue MCF7 or MDA-MB.231. The co-culture has been set up in presence or not of a multi-well insert that avoided the cell-to-cell contact (****P ≤ 0.0001). **e–f** Flow cytometry analysis of the pdASCs mitochondria fluorescence, into the recipient BCCs subset, after treatment with Antimycin A (100 nM), Cyt-B (2,5 uM), CCCP (5 uM) and MdiVi-1 (10 uM). **g** MTT assay for the evaluation of the viability of the cells used, in our co-culture system, after treatment with Cyt-B. For all the cell lines, the viability rate at 2,5 uM was higher than 85%. **h-i** Fluorescence microscopy of the co-culture with CellTracker-Blue pre-labelled MCF7 or **j-k** MDA-MB.231, for the detection of the F-Actin (FITC) and β-Tubulin (TRITC) in presence of Cyt-B (2,5 uM). The yellow arrows point-out the TNTs structures between the two kinds of cells (h-i) or the cytoplasmic F-actin aggregates (j-k) in both BCCs and pdASCs. (****P ≤ 0.0001)

MT was evaluated co-culturing pdASCs with luminal MCF7 and triple negative MDA-MB.231. pdASCs were isolated from human adipose tissue, on the basis of their phenotype: CD90^+^CD34^+^CD29^+^CD105^+^CD44^+^CD90^+^CD324^−^CD45^−^CD19^−^HLA-DR^−^ (Supplementary Fig. 1a) [19]. We showed that pdASCs mitochondria were transferred to BCCs through a complex communication network of TNTs. These are pdASCs scaffolded cell membrane projections, F-actin and β-tubulin positive, very rich in mitochondria along their entire length. Through these structures, pdASCs vehiculate their pre-labelled (MitoTracker-FITC/PE) mitochondria to pre-labelled (CellTracker-DAPI) BCCs. The exogenous pre-labelled mitochondria were observed in MCF-7 (Fig. 1a) and MDA-MB.231 (Fig. 1b), 24 h after the co-culture (Fig. 1a-b yellow arrows) and quantified by FACS analysis (Fig. 1c-d; Supplementary Fig. 1b-c) (****P ≤ 0.0001). The MT didn’t occur when the cells were physically separated by a multi-well cell culture inserts (Fig. 1c-d; Supplementary Fig. 1b-c).

Thereafter, exploring ways to inhibit MT, we treated both co-culture models with (i) Carbonyl Cyanide m-Chlorophenyl hydrazine (CCCP 5 µM) or (ii) Antimycin-A (0.1 µM), two different mitochondria respiration inhibitors, (iii) Mitochondrial division inhibitor-1 (MdiVi-1 10 µM), that inhibits Drp1, and (iv) Cytochalasin B (Cyt-B 2.5 µM), an actin polymerization inhibitor. We found that MdiVi-1 and Antimycin-A significantly reduced MT in MCF-7 (****P ≤ 0.0001). Furthermore, CCCP inhibited MT (****P ≤ 0.0001), but the effect was more evident in MCF-7 (%MFI = 13.6%) than in MDA-MB.231 (%MFI = 44.7%) (Fig. 1e-f; Supplementary Fig. 1d-e). Also, the actin polymerization inhibitor Cyt-B blocked MT (****P ≤ 0.0001) at sub-lethal doses (Fig. 1g), although MCF-7 were more sensitive (%MFI = 9.5% vs 48.7%, respectively).

Cyt-B very efficiently inhibited TNTs scaffolding (Fig. 1h-k), with dramatic effects on cell morphology. Indeed, cells’ structural collapsing was detected, with smaller appearance, more rounded shape, many membrane invaginations and accumulation of F-actin aggregates in the cytoplasm (Fig. 1j-k). Consequently, cells unable to scaffold the actin-based TNTs could not contact each other, and this impeded MT.

### MT occurs in patient-derived primary cultures and organoids

**Fig. 2:**
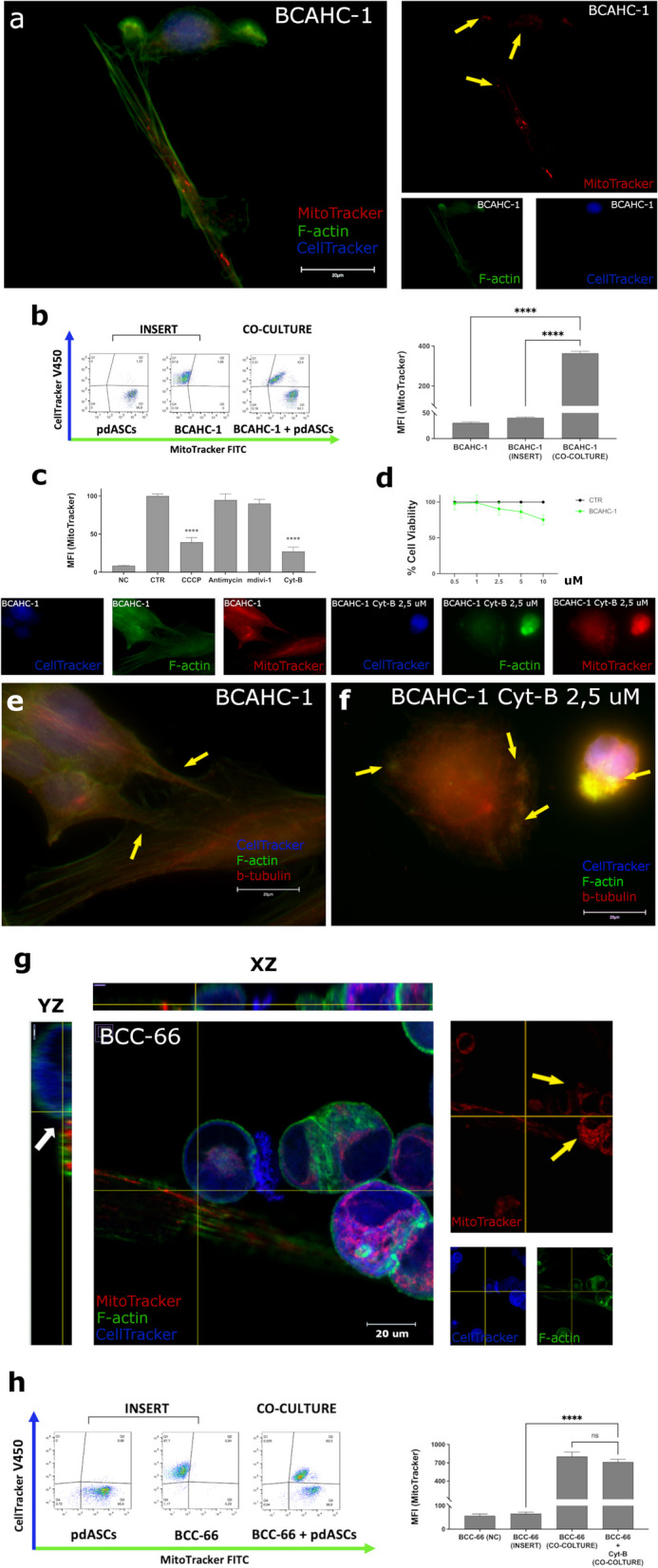
MT occurs both between pdASCs and human primary 2D and 3D cell models. **a** Fluorescence microscopy (magnification 100X) of pre-labelled BCAHC-1 (CellTracker-Blue) and pre-stained pdASCs (MitoTracker-Red) with F-Actin (Phalloidin-FITC). Yellow arrows point-out the pdASC mitochondria along the TNTs and into recipient BCAHC-1 cell. **b** Flow cytometry analysis of the cell fluorescence, for the quantization of the MT occurring from the pre-stained MitoTracker-FITC pdASCs to the pre-labelled CellTracker-Blue BCAHC-1. The co-culture has been set up in presence or not of a multi-well insert that avoided the cell-to-cell contact (****P ≤ 0.0001). **c** MT inhibition in co-culture between pdASCs and BCAHC-1. Flow cytometry analysis of the pdASCs mitochondria fluorescence, into the recipient BCAHC-1 subset, after treatment with Antimycin A (100 nM), Cyt-B (2,5 uM), CCCP (5 uM) and MdiVi-1 (10 uM) **d** MTT assay for the evaluation of the BCAHC-1 viability after treatment with Cyt-B. **e–f** Fluorescence microscopy of the co-culture with CellTracker-Blue pre-labelled BCAHC-1, for the detection of the F-Actin (FITC) and β-Tubulin (TRITC) in presence of Cyt-B (2,5 uM). The yellow arrows point-out the TNTs structures between the two kinds of cells (**e**) or the cytoplasmic F-actin aggregates (**f**) in both BCAHC-1 and pdASCs. (****P ≤ 0.0001). **g** Confocal microscopy 3D orthogonal reconstruction, z-stack technology of a 2D/3D hybrid coculture shows the pre-labelled BCC-66 (CellTracker-Blue) and pre-stained pdASCs (MitoTracker-Red) with F-Actin (Phalloidin-FITC). The white arrows in the YZ plane point-out that the pdASCs are in contact with the BCC-66 and the yellow arrow indicates the presence of exogenous mitochondria in the cytoplasm of BCC-66. **h** Flow cytometry analysis of the cell fluorescence, for the quantization of the MT occurring from the pre-stained MitoTracker-FITC pdASCs to the pre-labelled CellTracker-Blue BCC-66, also after treatment with Cyt-B (2 uM). The co-culture has been set up in presence or not of a multi-well insert that avoided the cell-to-cell contact

We verified that MT also occurs between pdASCs and the patient-derived breast cancer models BCAHC-1 and BCC-66. Interestingly, we observed the formation of TNTs between pdASCs and primary BCAHC-1 cells, through which mitochondria were vehiculated towards the BCAHC-1 cells (Fig. 2a, yellow arrows); the mechanism was also confirmed by FACS analysis (Fig. 2b; Supplementary Fig. 2a). Moreover, MT rate was significantly reduced by CCCP 5 µM (%MFI = 39,3%) and even more by Cyt-B (%MFI = 27,2%) at sub-lethal doses (Fig. 2c-d; Supplementary Fig. 2b). Notably, Cyt-B blocked TNTs scaffolding, structurally made of F-actin and β-Tubulin, as observed previously (Fig. 2e-f, yellow arrows). These profound morphological changes affected the cell–cell contact, significantly reducing MT.

Furthermore, we studied MT in BCC-66 PDOs isolated from a luminal breast cancer patient. Preliminarily, we showed that BCC-66 shared the same genomic profile of the original patient tissue (Supplementary Fig. 2c). According to our previous results, we built a hybrid 2D/3D co-culture between BCC-66, freed from BME, and pdASCs. BCC-66 were able to contact pdASCs, and massively acquired mitochondria from them (Fig. 2g, yellow arrows), although we did not capture TNTs formation. 3D orthogonal Z-stack image reconstruction confirmed that the adherent pdASCs directly contact BCC-66 in suspension (Fig. 2g, white arrow). MT was confirmed by FACS, but only in case of physical contact between the cells (****P ≤ 0.0001). Conversely, Cyt-B 2 µM did not significantly inhibit MT (Fig. 2h; Supplementary Fig. 2d), indicating that, in this hybrid 2D/3D co-culture, other mechanisms depending on physical contact could mediate MT.

### MitoCeption allows to increase mitochondria acquisition

**Fig. 3:**
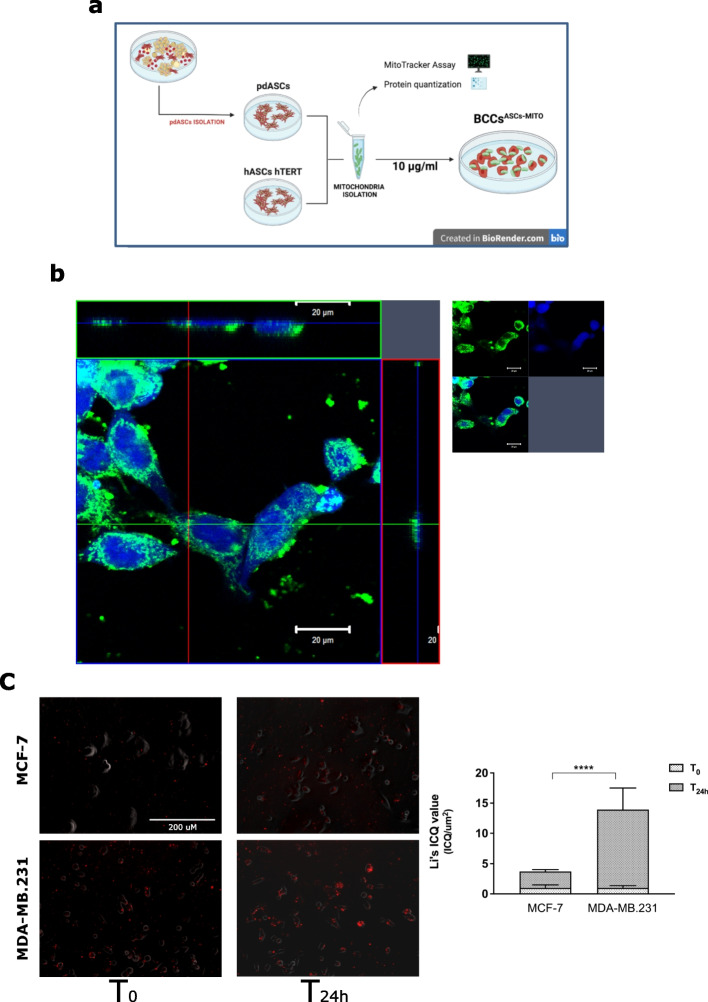
Construction and validation of MitoCeption model. **a** MCP assay workflow, from pdASCs/hASCs hTERT mitochondria-derived isolation to the forced engulfment into the BCCs. **b** Confocal microscopy 3D orthogonal reconstruction, with z-stack technology, of the pre-labelled CellTracker-Blue BCCs (MCF-7) after the MCP with MitoTracker-FITC pre-labelled pdASCs derived mitochondria **(c)** Fluorescence microscopy and spatial co-localization statistical analysis of BCCs and MitoTracker stained pdASCs derived mitochondria at time points 0 h and 24 h (Li’s ICQ value analysis normalized on the total cells area; ****P ≤ 0.0001)

To better distinguish the selective effect of exogenous mitochondria transferred to BCCs, we employed the MitoCeption (MCP), a procedure used to force mitochondria internalization, derived from a donor into a recipient cell that functionally engulf and retain them in its cytoplasm. According to *Caicedo *et al*.* [21], we isolated and characterized mitochondria from both pdASCs and hTERT immortalized hASCs; then we transferred them to MCF-7 or MDA-MB.231 cells (Fig. 3a). After 24 h, MCP was validated by Z-stack orthogonal view reconstruction of confocal images. Here, we identified pdASC pre-labelled mitochondria (MitoTracker-red) spatially distributed in the cytoplasm of the BCCs (CellTracker-DAPI) (Fig. 3b). MCP efficiency was evaluated verifying mitochondria internalization by statistical analysis of the co-localization between the transferred pdASCs mitochondria and the recipient cells (Fig. 3c). After 24 h (T_24h_) mitochondria co-localized with cells, as shown by analysis of the not-random spatial correlation. Moreover, MDA-MB.231 were significantly more able to intake exogenous mitochondria than MCF-7 (****P ≤ 0.0001) (Fig. 3c).

Taken together, our results show that MCP allows to increase the efficiency of mitochondria uptake and that MDA-MB.231 acquire significantly more mitochondria than MCF-7.

### ASCs-derived mitochondria potentiate BC multi-drug resistance

**Fig. 4:**
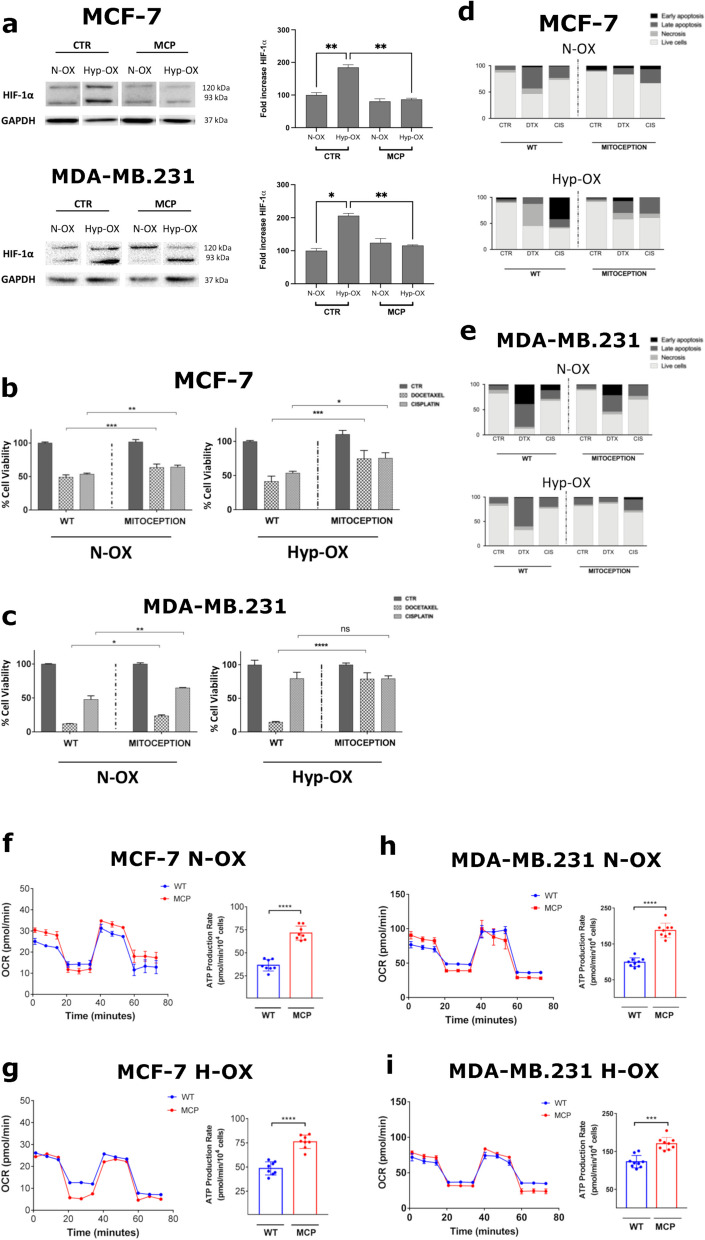
MCP increases BCCs viability under chemotherapy treatment and impacts on their metabolism, modifying mitochondrial respiration. **a** HIF-1α expression analysis in BCCs subjected to MCP, in N-OX and H-OX conditions. The MCP significantly reverted the up-regulation of HIF-1α in the BCCs after their stimulation with the chemical hypoxia inducer cobalt chloride (100 uM). **b-c** Cell viability and apoptosis assay **d-e** of the BCCs subjected to MCP in different oxygen conditions, after treatment with the chemotherapeutic drugs DTX (50 nM) or CIS (10 uM) (*P ≤ 0.05, **P ≤ 0.01, ***P ≤ 0.001; ****P ≤ 0.0001). **f-g** SeaHorse ATP rate assay for the analysis of the mitochondrial respiration in MCF-7 and (**h-i**) MDA-MB.231, 24 hours after the MCP, in different oxygen conditions. The histograms represent the ATP production rate (pmol/min), calculated during the oxygen consumption phase (****P ≤ 0.0001)

To functionally characterize MT effect, we investigated the antitumoral activity of different chemotherapy agents on BCCs carrying exogenous mitochondria. Considering that the MCP significantly downregulated HIF-1α protein expression in the recipient BCCs (Fig. 4a; Supplementary Fig. 3a-b), we decided to adopt this condition in our experiments.

Functionally, ASC-derived Mitochondria (ADM) significantly reduced the cytotoxicity of CIS (10 µM) and DTX (50 nM), after 24 h in both BCC lines (Fig. 4b-c).

Specifically, ADM was cytoprotective in MCF-7, towards both CIS (> 15%; **P ≤ 0.01) and DTX (> 11%; ***P ≤ 0.001) also in hypoxia (CIS > 33%; (*P ≤ 0.05-DTX > 22%; ***P ≤ 0.001) (Fig. 4b). Similarly, ADM protected MDA-MB.231 from CIS (> 15%; **P ≤ 0.01), with a milder effect in hypoxia (Fig. 4c). We obtained the same significant results towards DTX-induced cytotoxicity (*P ≤ 0.05), which was much stronger in hypoxia (> 60%; ****P ≤ 0.0001). Interestingly, the DTX resistance acquired by the ADM-carrying MCF-7, was due to their ability to modulate the apoptotic machinery, while we did not observe significant differences in the modulation of this mechanism after treatment with CIS (Fig. 4d; Supplementary Fig. 4a). Conversely, the cytoprotective effect mediated by ADM was very strong in hypoxia, towards both drugs. We showed the same effect also in ADM-carrying MDA-MB.231 (Fig. 4e; Supplementary Fig. 4b). Here, the DTX strongly promoted apoptosis, even in hypoxia, with a significant ADM-mediated reversion of the phenomenon.

Taken together, our results indicate that ADM increased MDR in BCCs preventing the induction of apoptosis, in both hypoxic and normoxic conditions.

### ASCs-derived mitochondria induce an increase of ATP production in BCCs

Since mitochondria play a pivotal role in energy homeostasis, we evaluated the effect of ADM on BCCs metabolism, via extracellular flux analysis. Here, ADM strongly upregulated ATP rate production, associated with mitochondrial respiration (Δ_ATP_). Indeed, in ADM-carrying MCF-7 we found a significantly higher basal (OCR > 18%) and maximal respiration (OCR > 10%), with a relative increase of ATP production rate (Δ_ATP_ > 35 pmol/min; ****P ≤ 0.0001) (Fig. 4f). Furthermore, we found a significant reduction of the spare respiration (OCR < 18%), mainly due to the increased basal oxygen consumption rate mediated by ADM. On the contrary, in hypoxia ADM did not significantly influence basal nor maximal respiration, however the total oxygen consumption capacity was raised, leading to a relevant increase of ATP production (Δ_ATP_ > 27 pmol/min; ****P ≤ 0.0001) (Fig. 4g). In MDA-MB.231 the effect of ADM on mitochondrial metabolism was very similar. In these cells, the basal respiration was significantly increased after MCP (OCR > 12%), without any significant difference on the maximal respiration (Fig. 4h). Even in this case, ADM significantly reduced the spare respiration (OCR < 18%), due to basal oxygen consumption rate increasing, which led to the doubling of ATP production rate (Δ_ATP_ > 90 pmol/min; ****P ≤ 0.0001). In hypoxic MDA-MB.231 the respiratory capacity as well as the basal respiration didn’t significantly change after MCP (Fig. 4i). Nevertheless, both these parameters increased (OCR > 12%) contributing to significantly enhancing the ATP production rate (Δ_ATP_ > 47 pmol/min; ****P ≤ 0.0001). Taken together, our results indicate that ADM led to an increase in ATP production.

### ASCs-derived mitochondria activate the ABC transporter-mediated drug efflux

As ATP-binding cassette (ABC) transporters are involved in BC MDR, we evaluated their role in our MCP model, focusing on P-gp, ABCG2 and ABCC1 [13] by fluorescence microscopy and FACS 8 h after the induction of hypoxia, and subsequent treatment with DOX. We chose DOX both to take advantage of its autofluorescence and because it is a backbone of chemotherapy treatment in BC [15].

**Fig. 5:**
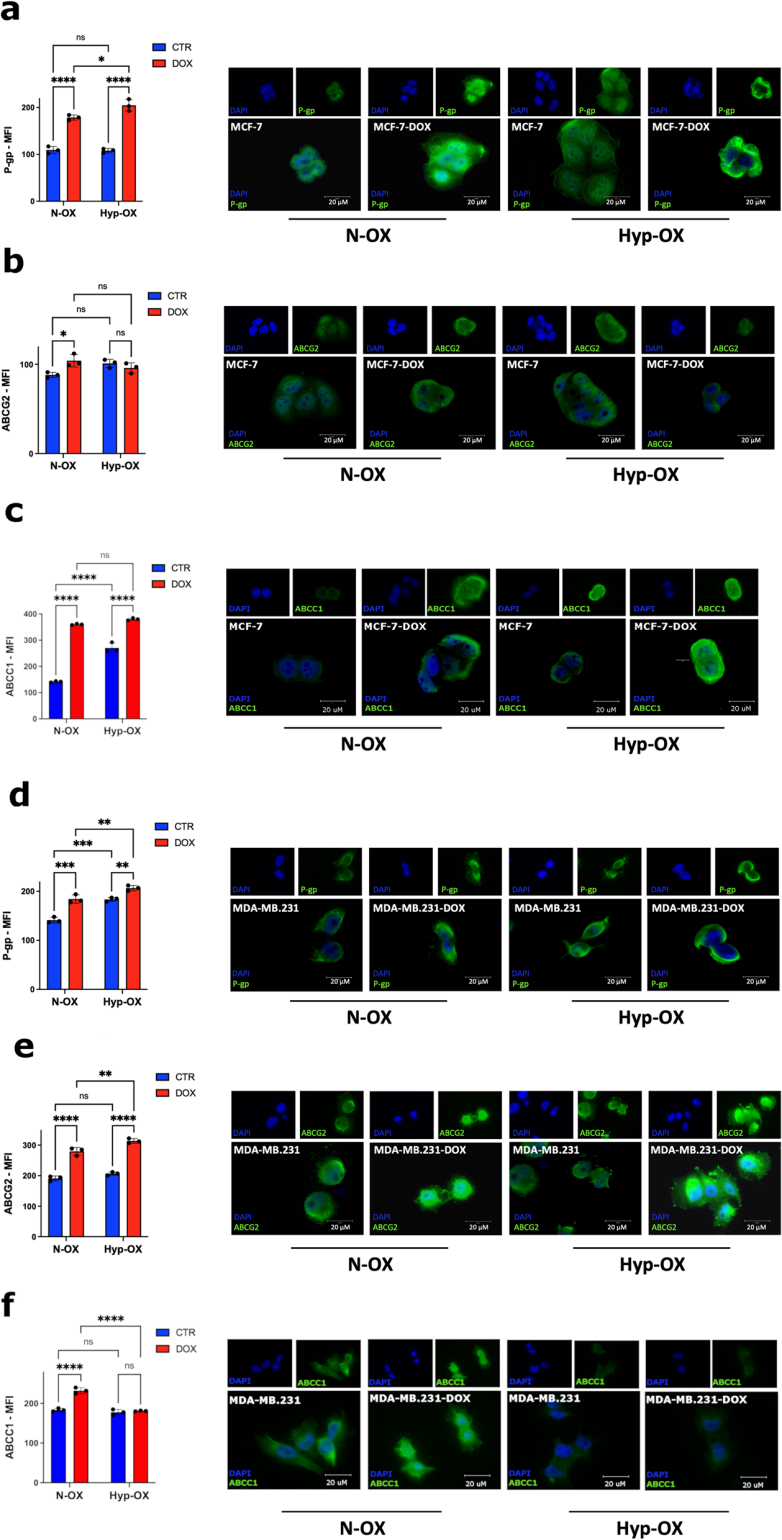
ABC transporters expression is influenced by oxygen conditions and is upregulated under doxorubicin treatment. Flow cytometry (plots) and fluorescent microscopy analysis for the quantitative and qualitative evaluation of the P-gp, ABCG2 and ABCC1 expression, in MCF-7 **(a-c)** and MDA-MB.231** (d-f)** 6 h after treatment with Doxorubicin (1 µM for MDA-MB.231; 2 µM for MCF-7), in N-OX or H-OX environment (*P ≤ 0.05, **P ≤ 0.01, ***P ≤ 0.001; ****P ≤ 0.0001)

In MCF-7, we observed that P-gp and ABCC1 expression increased after DOX treatment in both oxygen conditions, while ABCG2 increased after DOX only in normoxia (Fig. 5a-c; Supplementary Fig. 5). In MDA-MB.231 P-gp expression was increased after DOX (in both oxygen conditions) and in hypoxia only. ABCG2 was not influenced by hypoxia and increased only with DOX treatment in both oxygen conditions while ABCC1 only increased in normoxia after DOX treatment (Fig. 5d-f; Supplementary Fig. 5).

**Fig. 6:**
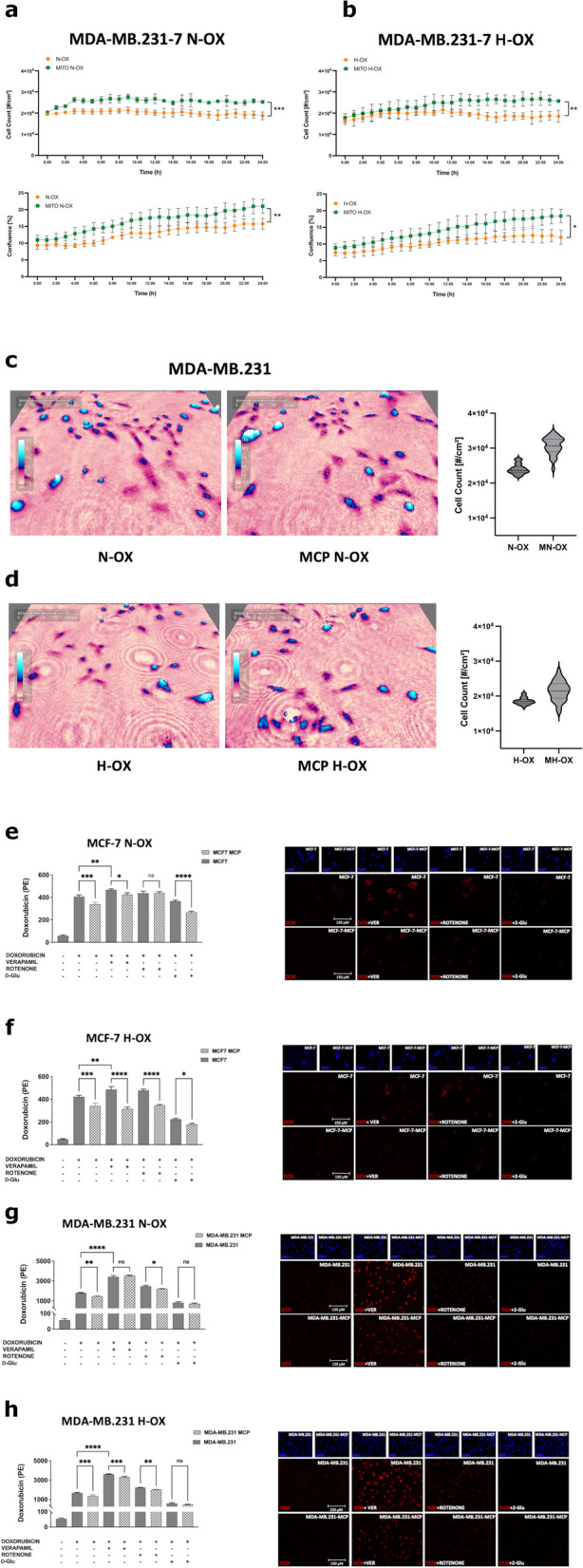
MCP is protective in MDA-MB.231 treated with Doxorubicin and promotes the ABC efflux activity in BCCs. In MDA-MB.231 **(a-b)** treated with DOX (1 µM), the cell counts and confluence significantly increase after MCP in both N-OX (#cells/cm^2^ = ***P ≤ 0.001, %confluence = (**P ≤ 0.01), and H-OX (#cells/cm.^2^ = ***P ≤ 0.01, %confluence = (**P ≤ 0.05) environments. Live time-lapse microscopy (capture from movie) **(c-d)** of MDA-MB.231, shows the cell population growth differences after MCP in both N-OX and H-OX conditions from T0 to T24 at a single well point. The inhibition of the ABC transporter with VER (5 µM) significantly blocks the efflux capacity of P-gp and ABCG2, independently of the cell line and the oxygen levels. The MCF-7 **(e–f)** and MDA-MB.231 **(g-h)** have been cultured in N-OX or H-OX micro-environment, and subsequently treated with DOX (1 µM for MDA-MB.231; 2 µM for MCF-7) together with the metabolic regulators 2-DG (50 mM), and ROTENONE (50 µM). After 24 h, the DOX cell retention has been evaluated quantitatively and qualitatively by flow cytometry and fluorescent microscopy (*P ≤ 0.05, **P ≤ 0.01, ***P ≤ 0.001; ****P ≤ 0.0001)

Subsequently, we focused our attention on the possible effect of ADM on ABC transporters activity. Preliminarily, we evaluated DOX cytotoxicity on BCCs after MCP. Indeed, it induced an increase of cell viability parameters monitored with Holomonitor (***P ≤ 0.001), also in hypoxia (**P ≤ 0.01) **(**Supplementary Fig. 6a-b). In MDA-MB.231, we achieved the same results in normoxia (#cells/cm^2^ = ***P ≤ 0.001; %confluence = **P ≤ 0.01) and hypoxia (#cells/cm^2^ = **P ≤ 0.01; %confluence = *P ≤ 0.05) (Fig. 6a-d). Thereafter, we evaluated DOX cytoplasmic retention by measuring the DOX_MFI_. As expected, the ABC-transporter inhibitor VER increased DOX cytoplasmic accumulation [23]. Nevertheless, ATP metabolism was crucial in regulating the activity of these transporters. Indeed, in both oxygen conditions, the ATP synthesis inhibitor Rotenone strongly reduced ABC transporters efflux capacity, and this effect was significantly reverted by D-Glu (Fig. 6e-h; Supplementary Fig. 7). These results highlight that ABC transporter activity is strictly dependent on ATP availability, independently from cell respiration.

In ADM-carrying MCF-7 we found a reduced DOX accumulation (DOX_MFI_ < 16%; ***P ≤ 0.001), even after treatment with VER (DOX_MFI_ < 9%; *P ≤ 0.05), not observed after treatment with Rotenone (Fig. 6e; Supplementary Fig. 7). Nevertheless, the administration of D-Glu again boosted the ADM-mediated effect (DOX_MFI_ < 27%; ****P ≤ 0.0001). In hypoxia MCP was much more effective. In this condition, we demonstrated that ADM increased the drug efflux rate (DOX_MFI_ < 18%; ***P ≤ 0.001), even in case of ABC-transporters inhibition with VER (DOX_MFI_ < 35%; ****P ≤ 0.0001). Interestingly, although the Rotenone-mediated blockade of ATP synthesis affected cell detoxification ability, ADM significantly reverted this condition (DOX_MFI_ < 27%; ****P ≤ 0.0001); the same effect was observed after treatment with D-Glu (DOX_MFI_ < 26%; ****P ≤ 0.05) (Fig. 6f; Supplementary Fig. 7).

In MDA-MB.231 we found that ADM drove DOX resistance. Specifically, ADM significantly reduced DOX accumulation (DOX_MFI_ < 18%; **P ≤ 0.01), while no significant effect was observed with VER. Again, the Rotenone mediated DOX accumulation was effectively counteracted by the MCP (*P ≤ 0.05). In MDA-MB.231 the effect of MCP was more evident in hypoxia. In this case, DOX cytoplasmic levels were significantly decreased (DOX_MFI_ < 18%; ***P ≤ 0.001), also after the administration of VER (DOX_MFI_ < 8%; ***P ≤ 0.001). Moreover, the modulation of ABC transporters activity by MCP was evident after treatment with rotenone (DOX_MFI_ < 10%; **P ≤ 0.01), but not with D-Glu (Fig. 6g-h; Supplementary Fig. 7).

Taking all these results into consideration, we showed that ADM activated ABC transporters into both MCF7 and MDA-MB.231 cell lines, leading to a significant reduction of drug accumulation within the cytoplasm and escape from chemotherapy induced cytotoxicity.

## Discussion

Recent studies demonstrated that BC development and progression as well as treatment response depends also on its complex micro-environment [24, 25]. MSCs are recruited to the site of tumor formation, where they promote a more aggressive phenotype, in terms of acquired/enriched stemness, chemoresistance [26] and distant dissemination [5]. ASCs influence BCCs through the secretion of cytokines, chemokines and/or growth factors involved in cell proliferation and migration, inflammation and angiogenesis [27]. Moreover, ASCs also interact with BCCs either activating an intense vesicular trafficking [28] or directly by “cell–cell” connections. In this spatial configuration, ASCs can interact with BCCs through different mechanisms, such as receptor/ligand interactions [29], cell–cell fusion processes [30, 31] or TNTs formation [32, 33]. Indeed, in a previous study, we showed that BCCs are able to maintain hASCs in a ‘stem state’ in vitro, while hASCs promote tumor angiogenesis in vivo, favoring tumor growth and aggressiveness [19].

Mitochondria are cytoplasmic organelles that provide numerous bioenergetic and biosynthetic processes, whose dynamics and activity are strictly regulated. They play a pivotal role in the maintenance of cancer cell homeostasis, during tumor progression, because of their ability to favor the adaptive cell response to adverse conditions, such as oxidative stress, hypoxic environment or chemotherapy induced cytotoxicity [34]. In cancer cells mitochondria switch to a more fused pattern [35, 36], can promote the activation of many anti-apoptotic pathways, also favoring a higher ATP production linked to the respiratory chain, thanks to an increased mitochondrial cristae density [37].

In this study, we demonstrated the occurrence of mitochondrial transfer from ASCs to BCCs, that promotes an adaptive metabolic response and chemoresistance in the recipient cells. We developed different 2D cocultures, employing commercial cell lines belonging to different immunophenotypes, pdASCs and also patient-derived breast cancer models. We highlighted the formation of a complex TNTs network between pdASCs and adherent BCCs (MCF-7, MDA-MB.231 and the primary BCAHC-1), showing that this occurs in both luminal and triple negative models, as well as in patient-derived models. These membrane projections exhibit highly variable morphology, in terms of thickness and length, and are full of mitochondria. Connection via TNTs allows the trafficking of macro-molecules and/or of entire organelles, in particular mitochondria, between MSCs and cancer cells [38–40]. Here we showed that this TNTs allows to transfer mitochondria from pdASCs to BCCs (MCF-7 and MDA-MB.231). In these models we showed that MT was dependent on TNT’s formation, as highlighted by its abolishing when disrupting actin polymerization. These achievements were confirmed also with the adherent patient-derived primary BC cell line BCAHC-1. Indeed, even in this co-culture model, primary BCCs acquired mitochondria from pdASC, in a process strictly related to cytoskeleton remodeling. Furthermore, Cytochalasin B was the only drug that affected MT in all 2D cell lines, while the other gave differential effects. Moreover, MT was confirmed also from pdASCs to BCC-66, a patient-derived organoid model developed in our laboratory, showing that the process occurs also in a more physiological context. Nevertheless, in this hybrid 2D/3D coculture, the TNTs scaffolding blockade did not significantly affect MT, demonstrating that in this complex spatial model it occurs through additional cell–cell contact-driven mechanisms that deserve further investigation.

From a functional point of view, we characterized the biological effect mediated by the acquisition of exogenous mitochondria. To better dissect this aspect from the background noise, due to the plethora of cell–cell interactions in co-culture, we decided to adopt the MCP [21]. Thanks to this approach, we showed that ADM uptake modifies the cell oxygen balance, completely reverting the hypoxic status. In fact, ADM impressively reduced the expression of HIF-1α in our models subjected to inducible hypoxia. According to these results, we evaluated the relationship between ADM and BC drug resistance, also considering that BCCs immunophenotype significantly influences their sensitivity to the different chemotherapies. Indeed, some authors demonstrated that ERα-positive cells, such as MCF-7 are less sensitive to anthracyclines than ERα-negative cells, such as MDA-MB.231 [41]. Furthermore, *Caicedo *et al., showed that the internalization of MSCs-derived mitochondria into BCCs led to an increased OXPHOS and acquisition of drug resistance [21, 42], showing that metabolic rewiring can influence drug response. In our study, ADM promoted a metabolic switch in recipient cells. Indeed, it significantly improved MCF-7 basal mitochondrial respiratory activity in normoxic as well hypoxic conditions, increasing the oxygen capacity and the relative OXPHOS. Conversely, MDA-MB.231, that prominently depend on glycolysis, showed a switch to OXPHOS, with a significant increase of ATP production, mainly in normoxic conditions.

Since MCP induced cancer cells to be more resistant to chemotherapy, and the ABC drug efflux transporters are among the main proteins involved in MDR [43], we investigated on a possible cause-effect relationship between the MCP-induced metabolic switch and ABC transporter functionality. We employed docetaxel and anthracyclines which are universally employed drugs for the treatment of breast cancer in all disease settings and subtypes according to international guidelines, and cisplatin as platinum salts are employed for triple negative breast cancer. Recently, a BCCs chemo-resistant sub-population was described, with an elevated mitochondrial respiratory capacity due to the loss of Methylation-Controlled J protein (MCJ), an endogenous negative regulator of mitochondrial activity. The ATP production boost, observed in these models, fueled the ABC transporters which increased their efflux ability [15]. In our model, we found high expression levels of P-gp, ABCG2 and ABCC1, the ABC transporter isoforms which are prominently involved in BC-MDR [18, 44, 45]. Some studies correlate P-gp high protein expression with a higher metastatic potential in triple negative BC, while ABCG2 over-expression is significantly linked with a better prognosis [46]. Indeed, we found a high basal expression of these transporters, especially in the triple negative MDA-MB.231, but also in luminal MCF7. Moreover, we confirmed that hypoxia and the pressure exerted by chemotherapy positively regulated the expression of these efflux pumps, as previously demonstrated [47].

Thereafter, taking advantage of our purer model developed thanks to MCP, we investigated the impact of MT on ABC transporters functionality. Interestingly, we found that ADM significantly increased the DOXO efflux ability of MCF-7 and MDA-MB.231 in both oxygen conditions, but with a stronger effect in hypoxia, a condition that is more representative of the real tumor oxygen status.

Specifically, we demonstrated that MCP metabolically boost the BC recipient cells, leading to the intracellular increasing of ATP, a direct molecular activator of the ABC transporters. These efflux pumps play a key role in the BCCs MDR, as demonstrated in our models, where we found a strongly increased intracellular DOX retention in both oxygen conditions, by selectively blocking ABC receptors with VER [48].

**Fig. 7:**
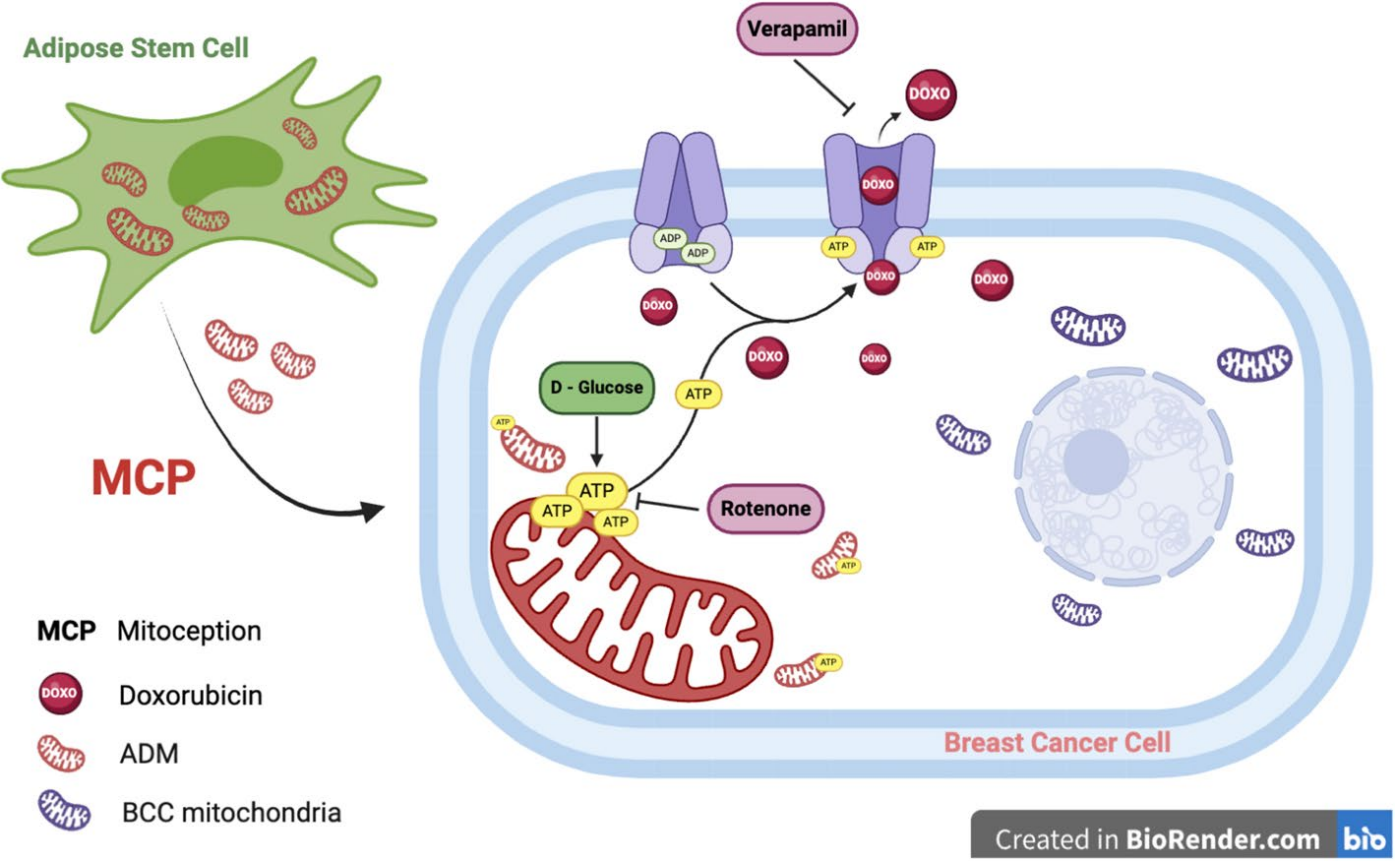
Proposed model of ABC transporter regulatory mechanism after ADM uptake

Our results show that the increased ATP generated after ADM uptake is the key modulator of the ABC receptor efflux in BCCs. Indeed, when we blocked ATP synthesis with rotenone, we saw an increase of DOX cytoplasmic retention, indicating a downregulation of ABC transporters function. On the other side, the administration of D-Glu reversed this inhibition, reactivating ABC efflux activity (Fig. 7).

## Conclusions

In conclusion, we demonstrate for the first time that MT promotes extensive BCCs changes leading to a more resistant phenotype. We discovered that pdASCs interact with BCCs, donating their own mitochondria. This MT is strictly related to cell structure remodeling, that allows the physical cell–cell contact. This mechanism drives many functional effects in the recipient cancer cell, that benefit from this process. Indeed, we showed that these cells improved their adaptive response to the extracellular environment, increased their mitochondrial respiration and the relative OXPHOS and upregulated ABC transporter activity, thereby acquiring a phenotype that better escape from anticancer therapies. Intriguingly, we also demonstrated the MT in more accurate and translational in vitro models that reproduce some key aspects of the tissue of origin. Furthermore, we show for the first time that inhibiting MT could effectively restore sensitivity to chemotherapy making it a new potential target to develop innovative treatment strategies. Further studies are needed to better elucidate the occurrence of alternative ways for cancer cells to attract exogenous mitochondria, but this study opens the way to new treatments for breast cancer, either by blocking the MT mechanisms from ASCs to BCCs or by inhibiting the interaction between mesenchymal stem cells and tumor cells.

### Supplementary Information

Additional file 1: Figure S1 a Flow cytometry histograms related to the pdASCs markers expression. b-c Flow cytometry histograms related to the evaluation of the mitochondrial transfer, from the pdASCs to MCF-7 and MDA-MB in normal conditions, (d-e) and after treatment with the mitochondrial activity inhibitors CCCP, Antimycin A, MidiVi-1 or with the actin polymerization inhibitor Cyt-B.

Additional file 2: Figure S2 Flow cytometry histograms related to the evaluation of the mitochondrial transfer, from the pdASCs to the BCAHC-1 in (a) normal conditions, and (b) after treatment with the mitochondrial activity inhibitors CCCP, Antimycin A, MidiVi-1 or with the actin polymerization inhibitor Cyt-B. (c) Comparative analysis of BCC66 primary line molecular profile compared to a BT66 FFPE tumour sample. The table shows the statistically significant mutations, in the same locus, with comparable allele frequencies. All the other genes analysed included in the 3 panels adopted were not graphically reported as they were not mutated (wild-type).

Additional file 3: Figure S3 a-b HIF 1 alpha expression analysis for BCCs in N-OX and H-OX conditions with and without MCP.

Additional file 4: Figure S4 a-b Annexin V-PI assay for the evaluation of the cell death quality, in BCCs treated with chemotherapy agents, under different oxygen conditions.

Additional file 5: Figure S5 a-f Flow cytometry analysis for the evaluation of the expression levels of P-gp and ABCG2, on BCCs treated with DOX under different oxygen conditions.

Additional file 6: Figure S6 a-b Kinetic dose–response assay for the evaluation of the cytotoxic effect of DOX-treated MCF-7 before and after MCP, under different oxygen conditions.

Additional file 7: Figure S7 a-d Flow cytometry histograms related to the cytoplasmic retention of the doxorubicin in the BCCs, subjected to MCP, in different oxygen conditions, after treatment with DOX, VER, ROTENONE and D-Glu.

Additional file 8: Supplementary Table 1. Breast cancer organoids media components.

Additional file 9: Supplementary Table 2. AmpliSeq Cancer Hotspot Panel v2.

Additional file 10: Supplementary Table 3. 2X Oncomine™ Focus DNA Assay.

Additional file 11: Supplementary Table 4. Ion AmpliSeq™ Custom Gastric Panel.

## Data Availability

All data generated or analyzed in this study are included in this article and its supplementary information files. Sequencing data will be freely provided upon request.

## Abbreviations

MT: Mitochondrial transfer
ASCs: Adipose stem cells
BCCs: Breast cancer cells
TNTs: Tunneling nano tubes
MDR: Multi-drug resistance
BC: Breast cancer
BCME: Breast cancer microenvironment
MSCs: Mesenchymal stem cells
EMT: Epithelial-to-mesenchymal transition
CSCs: Cancer stem cells
EVs: Extracellular vesicles
ROS: Reactive oxygen species
ATP: Adenosine-5’-Triphosphates
ABC: ATP-binding cassette
FBS: Fetal bovine serum
pdASCs: Patient derived-ASCs
MFI: Median fluorescence intensity
MCP: MitoCeption
OCR: Oxygen consumption rates
ECL: Enhanced chemiluminescence
MFI_FITC_: FITC median fluorescence intensity
MdiVi-1: Mitochondrial division inhibitor-1
Cyt-B: Cytochalasin B
CTR: Untreated cells
ADM: ASC-derived mitochondria
CIS: Cisplatin
DTX: Docetaxel
DOX: Doxorubicin
VER: Verapamil
MCJ: Methylation-controlled J protein
