# Supplementary figures and images for: Mitochondrial transfer from Adipose stem cells to breast cancer cells drives multi-drug resistance
Source: J Exp Clin Cancer Res. 2024 Jun 14;43:166. doi: 10.1186/s13046-024-03087-8 (PMC11177397; doi:10.1186/s13046-024-03087-8)

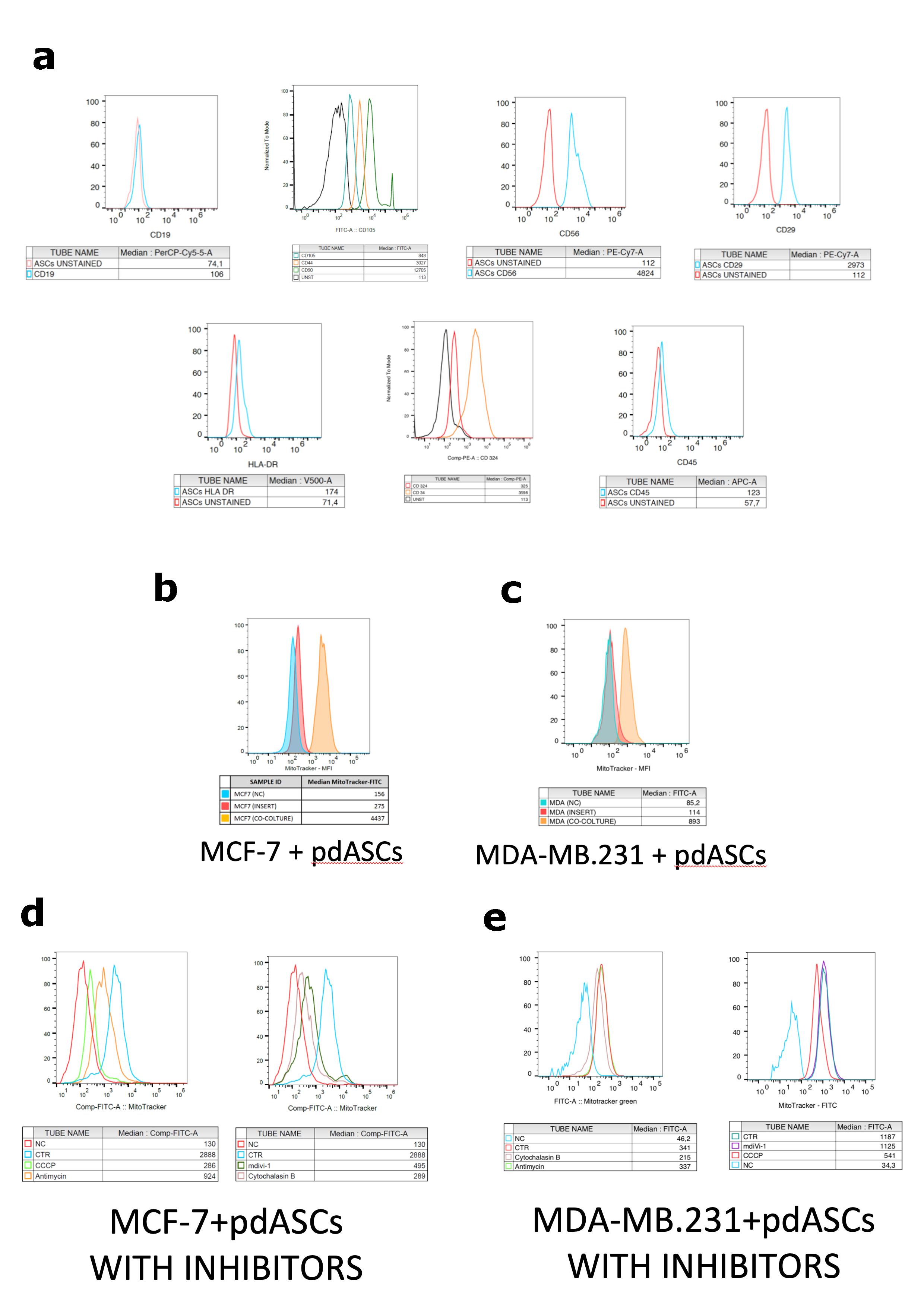

Supplement: Supplementary file 1 — Additional file 1: Figure S1 a Flow cytometry histograms related to the pdASCs markers expression. b-c Flow cytometry histograms related to the evaluation of the mitochondrial transfer, from the pdASCs to MCF-7 and MDA-MB in normal conditions, (d-e) and after treatment with the mitochondrial activity inhibitors CCCP, Antimycin A, MidiVi-1 or with the actin polymerization inhibitor Cyt-B. [file 13046_2024_3087_MOESM1_ESM.tiff]

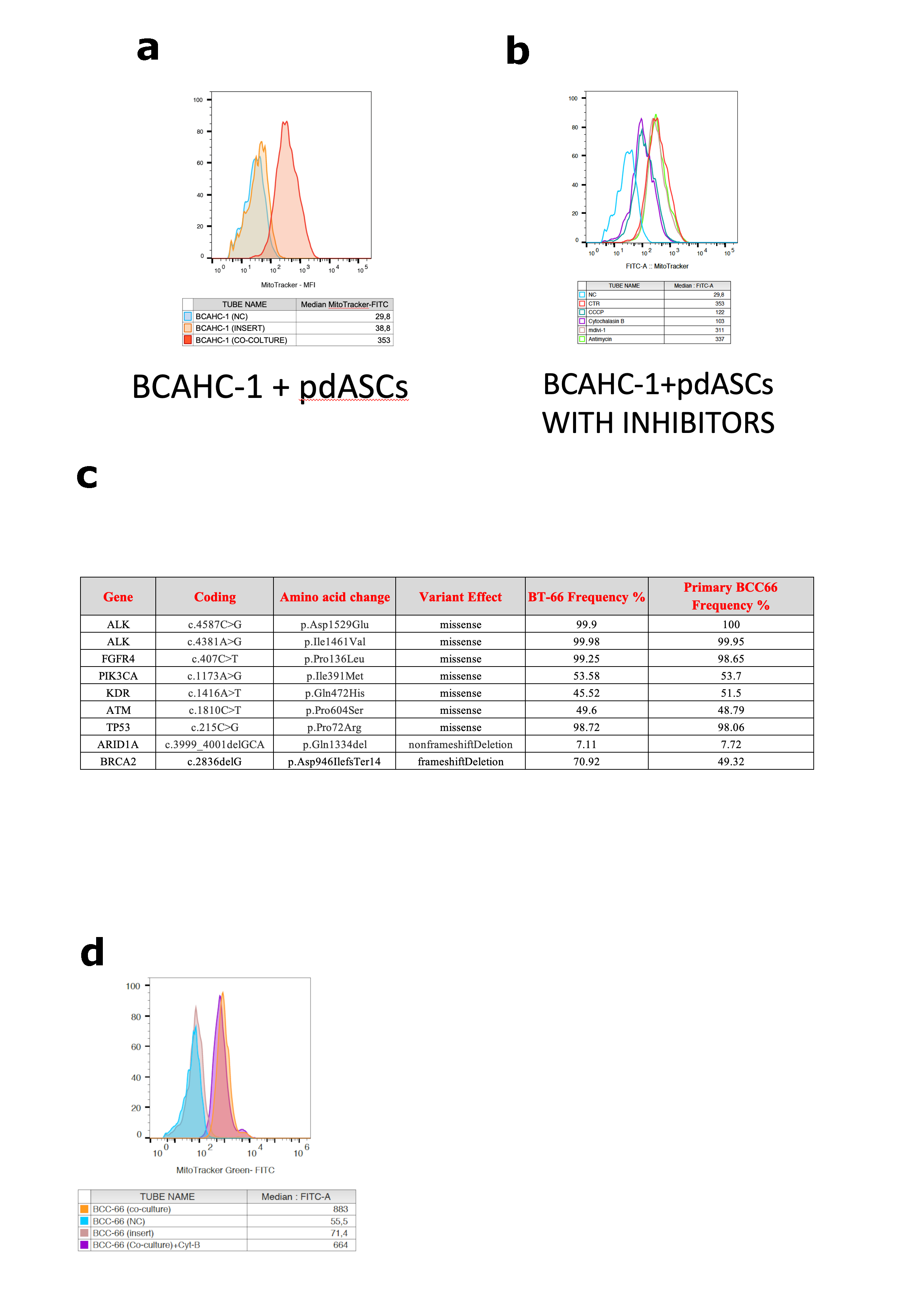

Supplement: Supplementary file 2 — Additional file 2: Figure S2 Flow cytometry histograms related to the evaluation of the mitochondrial transfer, from the pdASCs to the BCAHC-1 in (a) normal conditions, and (b) after treatment with the mitochondrial activity inhibitors CCCP, Antimycin A, MidiVi-1 or with the actin polymerization inhibitor Cyt-B. (c) Comparative analysis of BCC66 primary line molecular profile compared to a BT66 FFPE tumour sample. The table shows the statistically significant mutations, in the same locus, with comparable allele frequencies. All the other genes analysed included in the 3 panels adopted were not graphically reported as they were not mutated (wild-type). [file 13046_2024_3087_MOESM2_ESM.tif]

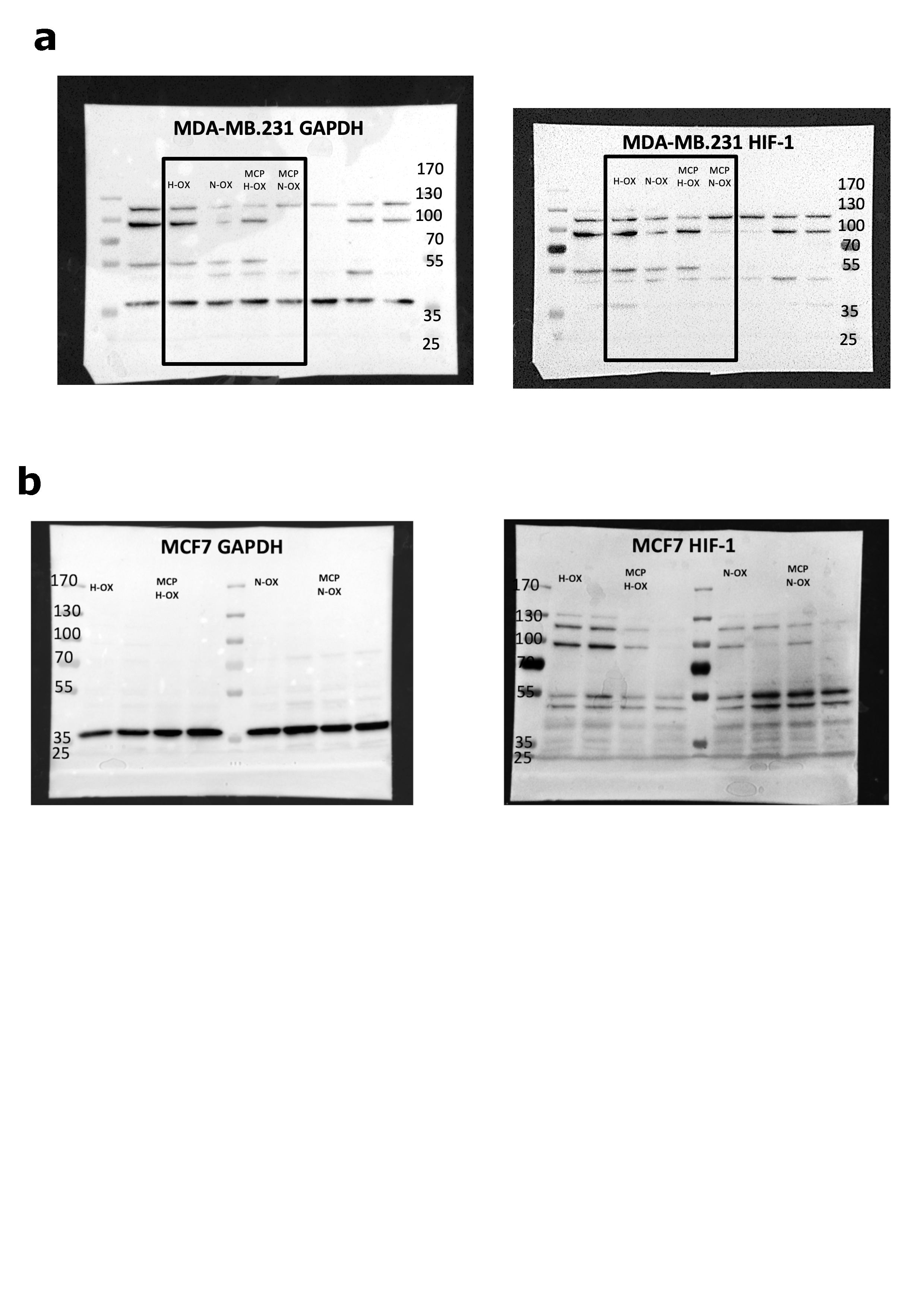

Supplement: Supplementary file 3 — Additional file 3: Figure S3 a-b HIF 1 alpha expression analysis for BCCs in N-OX and H-OX conditions with and without MCP. [file 13046_2024_3087_MOESM3_ESM.tiff]

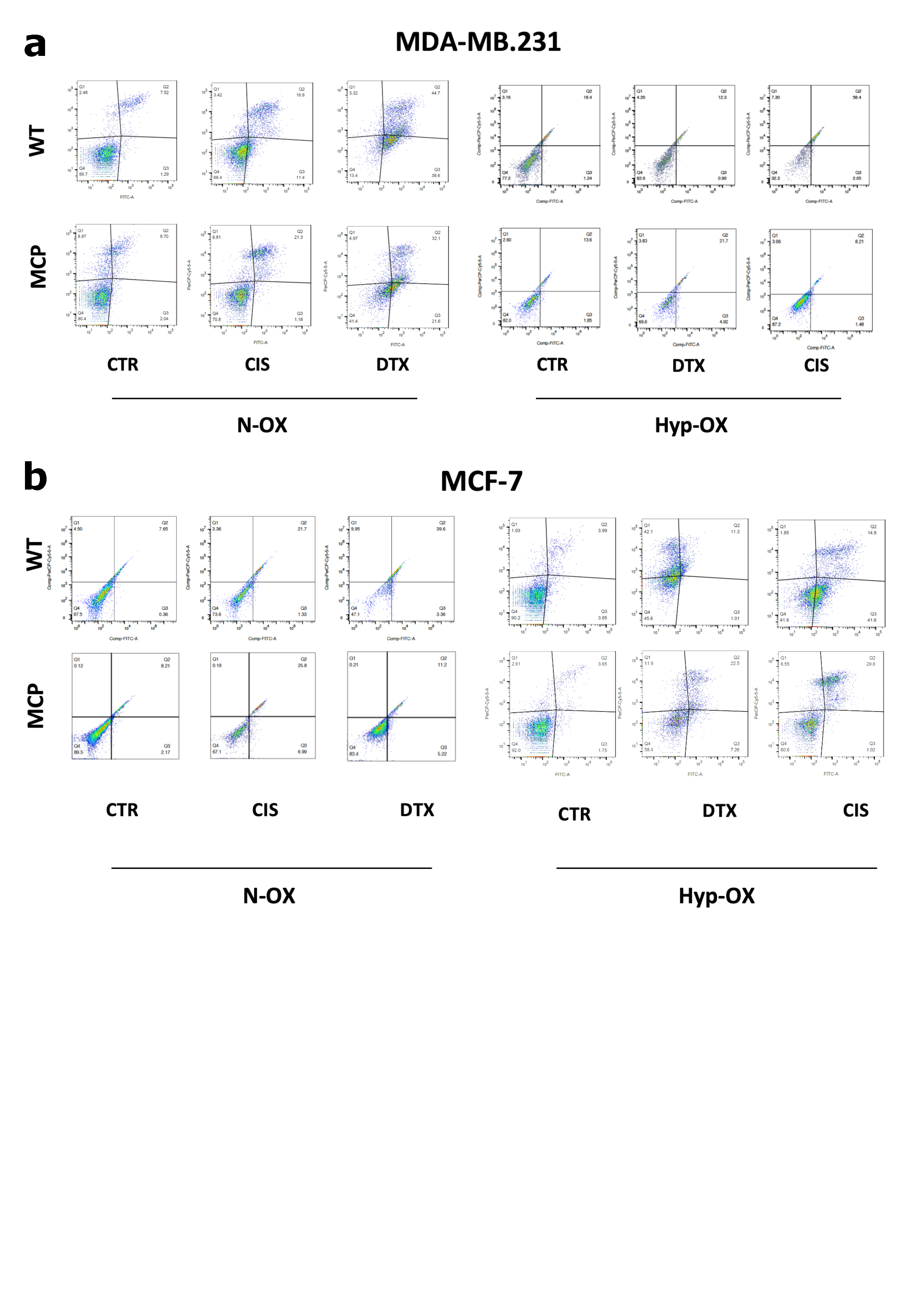

Supplement: Supplementary file 4 — Additional file 4: Figure S4 a-b Annexin V-PI assay for the evaluation of the cell death quality, in BCCs treated with chemotherapy agents, under different oxygen conditions. [file 13046_2024_3087_MOESM4_ESM.tiff]

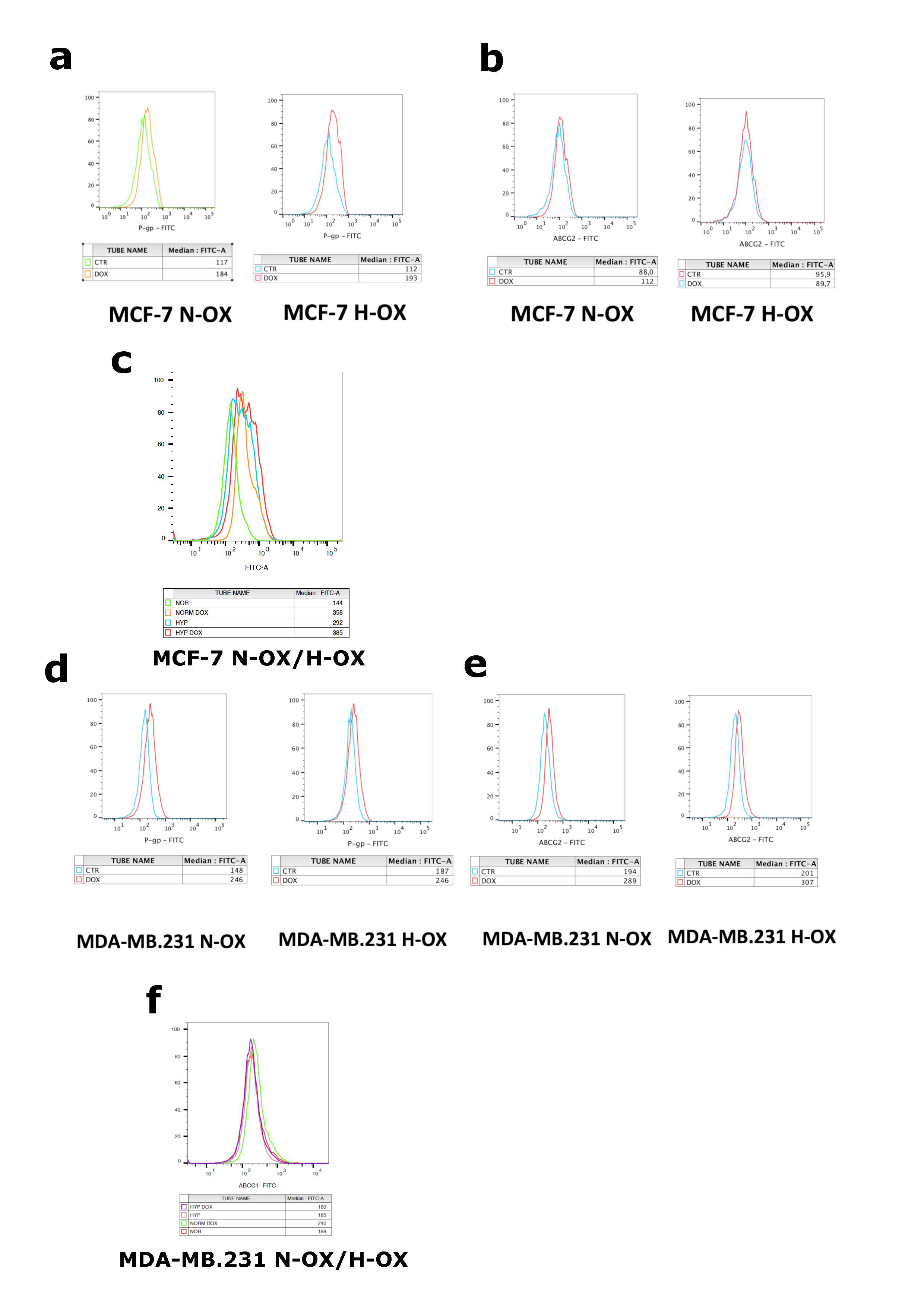

Supplement: Supplementary file 5 — Additional file 5: Figure S5 a-f Flow cytometry analysis for the evaluation of the expression levels of P-gp and ABCG2, on BCCs treated with DOX under different oxygen conditions. [file 13046_2024_3087_MOESM5_ESM.tiff]

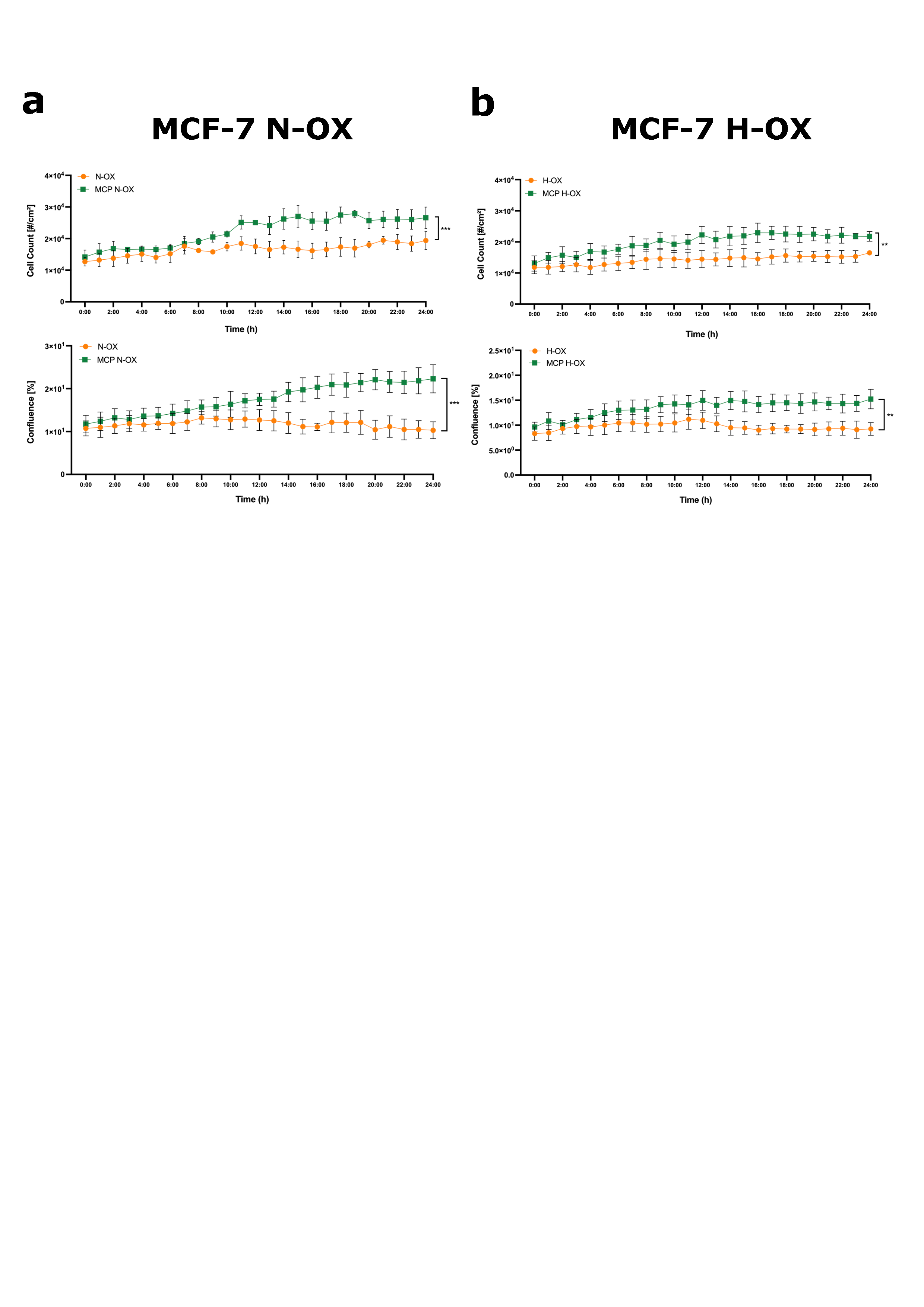

Supplement: Supplementary file 6 — Additional file 6: Figure S6 a-b Kinetic dose–response assay for the evaluation of the cytotoxic effect of DOX-treated MCF-7 before and after MCP, under different oxygen conditions. [file 13046_2024_3087_MOESM6_ESM.tiff]

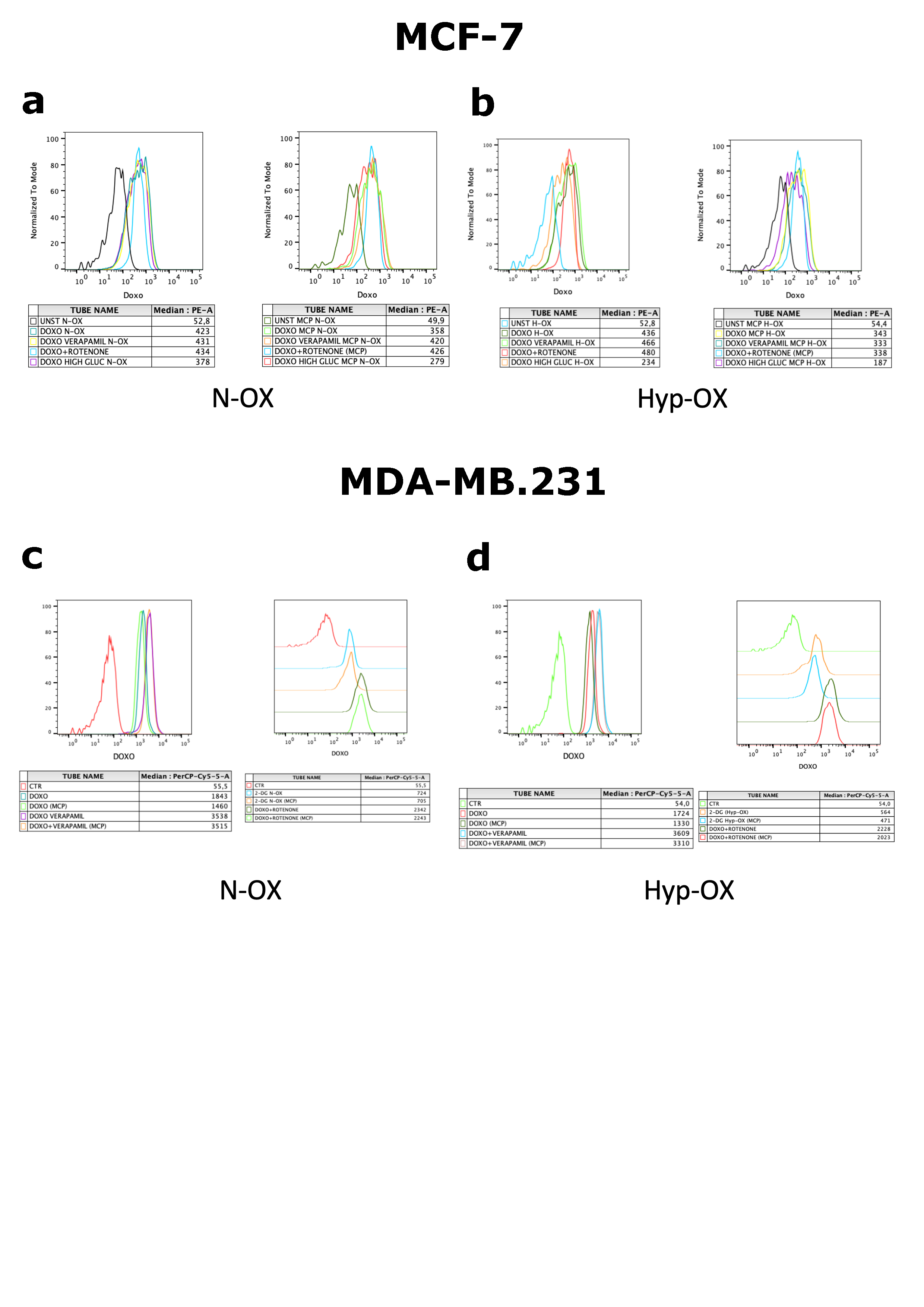

Supplement: Supplementary file 7 — Additional file 7: Figure S7 a-d Flow cytometry histograms related to the cytoplasmic retention of the doxorubicin in the BCCs, subjected to MCP, in different oxygen conditions, after treatment with DOX, VER, ROTENONE and D-Glu. [file 13046_2024_3087_MOESM7_ESM.tiff]
